# Nitric oxide signal is required for glutathione-induced enhancement of photosynthesis in salt-stressed S*olanum lycopersicum* L

**DOI:** 10.3389/fpls.2024.1413653

**Published:** 2024-06-17

**Authors:** Yundan Cong, Xianjun Chen, Jiayi Xing, Xuezhen Li, Shengqun Pang, Huiying Liu

**Affiliations:** ^1^ Department of Horticulture, Agricultural College, Shihezi University, Shihezi, Xinjiang, China; ^2^ Key Laboratory of Special Fruits and Vegetables Cultivation Physiology and Germplasm Resources Utilization of Xinjiang Production and Contruction Crops, Shihezi, Xinjiang, China; ^3^ School of Life and Health Science, Kaili University, Kaili, Guizhou, China

**Keywords:** Calvin cycle, fast OJIP fluorescence rise, glutathione, nitric oxide, salt stress, tomato

## Abstract

Reduced glutathione (γ-glutamyl-cysteinyl-glycine, GSH), the primary non-protein sulfhydryl group in organisms, plays a pivotal role in the plant salt stress response. This study aimed to explore the impact of GSH on the photosynthetic apparatus, and carbon assimilation in tomato plants under salt stress, and then investigate the role of nitric oxide (NO) in this process. The investigation involved foliar application of 5 mM GSH, 0.1% (w/v) hemoglobin (Hb, a nitric oxide scavenger), and GSH+Hb on the endogenous NO levels, rapid chlorophyll fluorescence, enzyme activities, and gene expression related to the Calvin cycle in tomato seedlings (*Solanum lycopersicum* L. cv. ‘Zhongshu No. 4’) subjected short-term salt stress (100 mM NaCl) for 24, 48 and 72 hours. GSH treatment notably boosted nitrate reductase (NR) and NO synthase (NOS) activities, elevating endogenous NO signaling in salt-stressed tomato seedling leaves. It also mitigated chlorophyll fluorescence (OJIP) curve distortion and damage to the oxygen-evolving complex (OEC) induced by salt stress. Furthermore, GSH improved photosystem II (PSII) electron transfer efficiency, reduced Q_A_^-^ accumulation, and countered salt stress effects on photosystem I (PSI) redox properties, enhancing the light energy absorption index (PI_abs_). Additionally, GSH enhanced key enzyme activities in the Calvin cycle and upregulated their genes. Exogenous GSH optimized PSII energy utilization via endogenous NO, safeguarded the photosynthetic reaction center, improved photochemical and energy efficiency, and boosted carbon assimilation, ultimately enhancing net photosynthetic efficiency (P_n_) in salt-stressed tomato seedling leaves. Conversely, Hb hindered P_n_ reduction and NO signaling under salt stress and weakened the positive effects of GSH on NO levels, photosynthetic apparatus, and carbon assimilation in tomato plants. Thus, the positive regulation of photosynthesis in tomato seedlings under salt stress by GSH requires the involvement of NO.

## 1 Introduction

China is the world’s largest horticultural country, Nevertheless, the escalation of secondary soil salinization, attributed to issues like high cropping intensity, excessive fertilization, and unique water transport mechanisms during production, poses a significant hurdle to the development and productivity of greenhouse crops in China (Zhu, 2016). Plant growth and development are closely linked to their photosynthesis, which is very sensitive to salt stress (Yang et al., 2022). Under salt stress, the photochemical efficiency of the plant photosystem is reduced, leading to overexcitation of the light-trapping antennae and the generation of oxidative stress. Sustaining photosynthesis under salt stress conditions hinges on regulating the photosynthetic apparatus. Moreover, salt stress induces photochemical bursts and diminishes photosynthetic quantum efficiency (Zushi and Matsuzoe, 2017), salt stress can also limit photosynthesis in plants by inactivating CO_2_ assimilating enzymes (Li et al., 2022a), resulting in reduced products of photosynthetic carbon assimilation and, in severe instances, potential plant death (Fatma et al., 2014). Tomato (*Solanum lycopersicum* L.) is a significant vegetable crop in horticulture, exhibiting moderate salt tolerance but some sensitivity to salt stress (Xu et al., 2023). Hence, exploring the photosynthetic acclimation mechanism of tomatoes under salt stress is valuable for improving their salt tolerance, reducing the damage caused by salt stress, breeding a resilient crop. Rapid chlorophyll fluorescence-induced kinetic analysis (JIP-test) is an analytical method established on the basis of biofilm energy flow. The JIP-test allows for rapid diagnosis of damage to the structure of the photosynthetic apparatus of a plant before it develops a visible pheno (Baker, 2008; Timilsina et al., 2022).

Reduced glutathione (γ-glutamyl-cysteinyl-glycine, GSH), a redox-active molecule, plays a pivotal role in the plant stress responses through redox pairs (GSH/GSSG) in the ascorbate-glutathione (AsA-GSH) cycle (Yao et al., 2021), glutathione, thioredoxin system, and other pathways. GSH can enhance the ability of plants to cope with challenges by protecting photosystem II by reducing reactive oxygen species (ROS) production (Zhou et al., 2018), protecting photosystem components, and increasing net photosynthetic rate (Mueller-Schuessele et al., 2020). Additionally, GSH collaborates with melatonin (Goodarzi et al., 2020), ascorbic acid, proline, or redox molecules (Semida et al., 2021) to collectively engage in stress-induced signaling pathways.

Nitric oxide (NO), a crucial bioactive plant molecule, regulates plant defense responses to various negative stressors. NO can effectively alleviate chlorophyll degradation, damage to the photosynthetic apparatus, reduced photochemical activity, and light energy conversion efficiency of PSII under stress, this enhances the plants’ photosynthetic adaptation (Murchie and Niyogi, 2011; Zhou et al., 2018).To date, more studies have been reported on the involvement of GSH in NO-controlled mitigation of adversity stress (Gautam et al., 2021; Wu et al., 2021; Xu et al., 2023). S-nitrosoglutathione (GSNO), a NO transporter and donor in plants, acts as a link between ROS and reactive nitrogen (RNS) signaling pathways, playing an important role in various plant signaling and defense reactions (Khan et al., 2023). GSNO/NO regulates the antioxidant system in different abiotic stress environments and improves plant resistance, suggesting that GSH participates in the NO signaling pathway to combat abiotic stresses (Hasan et al., 2016). The previous study demonstrated a synergistic role of NO and GSH in enhancing PSII activity and PSI transduction in cucumber leaves exposed to low-temperature stress (Yang et al., 2023). Currently, only a few studies have reported NO contributions to reducing abiotic stress-induced damage in plants via GSH (Hasan et al., 2016). Alamri et al. (2021) reported that GSH mitigation of arsenic toxicity in *Solanum melongena* requires the involvement of endogenous NO.

The authors’ previous studies demonstrated that exogenous GSH protects the photosynthetic apparatus from oxidative damage, increases PSII efficiency, and balances the uneven distribution of light energy by maintaining chloroplast redox balance and increasing ROS absorption capacity. GSH therefore effectively mitigates the inhibitory effects of salt on the growth and photosynthesis of tomato seedlings (Zhou et al., 2018). Furthermore, in another of the authors’ studies, the application of the NO scavenger Hb (hemoglobin bovine) reduced the exogenous GSH role in inducing endogenous NO production and increasing the antioxidant capacity of tomato (Wen et al., 2018). NO involvement was therefore hypothesized in the modulation of salt acclimatization by exogenous GSH. However, the role of exogenous GSH in regulating the structure and function of the photosynthetic and carbon assimilation systems in tomatoes under salinity stress, as well as the potential involvement of NO in the photoadaptative mechanism by which GSH alleviates salt stress, is still unclear. Therefore, the present study investigated the role of GSH in photosynthetic efficiency, the kinetic properties of rapid chlorophyll fluorescence induction, and carbon assimilation capacity in salt-stressed tomato seedlings via exogenous application of GSH, Hb, and Hb + GSH. NO involvement in the photosynthetic acclimatization induced by GSH was then detected and elucidated.

## 2 Materials and methods

### 2.1 Growing conditions and treatments

The hydroponics experiment was conducted in a greenhouse at Shihezi University, Xinjiang Uygur Autonomous Region,China. Tomato seeds (*Solanum lycopersicum* L. cv. ‘Zhongshu No. 4’) obtained from Shihezi Yaxin Seeds, China, Soak tomato seeds in warm water at 65°C for 30 minutes, stirring constantly during this time to allow them to fully soak. Subsequently, the seeds were gently rinsed with deionized water and planted in a 2:1 (v/v) charcoal and vermiculite mixture. The seedlings were grown under ambient conditions of 20–26°C, 40–60% relative humidity (RH) and approximately 500 µmol·photons·m^−2^·s^−1^ light intensity at noon. Upon reaching three fully mature leaves, uniform and healthy tomato seedlings were selected, roots were cleaned, and then transferred to a hydroponic system with a foam lid. The system was filled with 10 L of Hoagland’s nutrient solution (pH 6.2) prepared using demineralized water.

After allowing tomato seedlings to pre-culture for 7 days, five treatments were employed in the study (**Table 1**). The NaCl was added to the Hoagland nutrient solution, and GSH and Hb were sprayed on the leaves at 10:00 a.m every day. The concentrations of NaCl, GSH and Hb were established based on the pre-test results (Zhou et al., 2017; Wen et al., 2018). The research adopted a randomized block design with three replicates. Fully expanded functional young tomato seedling leaves in the third down from the growth point were chosen and sampled (with veins removed) at 24, 48, 72 hours post-treatment. The harvested leaves were rapidly frozen in liquid nitrogen and stored at -80°C for subsequent enzyme activity and gene expression assessments.

**Table 1:** Experimental protocols employed to investigate the impact of salt stress on tomato seedlings.

Treatment	Control	NaCl	NaCl+GSH	NaCl+Hb	NaCl+Hb+GSH
NaCl	0 mM	100 mM	100 mM	100 mM	100 mM
GSH	—	—	5 mM	—	5 mM
Hb	—	—	—	0.1% (W/V)	0.1% (W/V)

100 mM NaCl is added to the Hogland nutrient solution. Hb, GSH Spray on the leaves.

### 2.2 Visualization and quantification of endogenous NO signal

After 24, 48 and 72 hours of treatment, tomato leaves were sectioned into 5 x 5 mm^2^ pieces a dark-incubated in Tris-HCl buffer (10 mM Tris, containing 10 μM DAF-2DA, pH 7.4) for 30 min at 25°C. The excitation and emission wavelengths were 495 and 515 nm, correspondingly. Rinse 2–3 times with fluorescent dye-free buffer to remove excess fluorescent probe. Samples were examined utilizing a ZEISS LSM 510 META laser confocal scanning microscope (Zeiss, Germany). The mean fluorescence intensity of each field of view was quantified using the LSM510 software supplied with the instrument (Li et al., 2022a).

### 2.3 Assessment of nitrate reductase and NO Synthase activities

NR and NOS activities in tomato seedling leaves were measured using specific kits (Nanjing JianCheng Bio, China).

### 2.4 Determination of photosynthetic gas exchange parameters

Functional tomato leaves were harvested 24, 48, 72 hours post-treatment. Photosynthetic parameters were assessed with the CIRAS-3 photosynthesis system (PP Systems, USA) (Diao et al., 2014).

### 2.5 Polyphasic fluorescence transients and JIP-test parameters

Chlorophyll-a fluorescence kinetics (OJIP curves) and modulated reflectance kinetics at 820 nm (I/Io curves) were measured in tomato plants under various treatments using a multifunctional plant efficiency analyzer (Hansatech Instrument Ltd, Lynn, UK). After a two-hour dark acclimatization period, the leaves were exposed to saturating pulsed light (3,000 μmol photons m^-2^ S^-1^). The recorded fluorescence signals ranged from 0.01 ms to 3 S. The average OJIP measurement value of the OJIP measurement was calculated for the leaves (three tomato plants per treatment, n=3) in each treatment, and the fast OJIP curve was plotted. The spider plot data were presented as C_test_/C_Control_, where C_test_ and C_Control_ represented the data from the treatment and control groups, respectively. The JIP-test analysis of the OJIP curve provided parameters listed in **Table 2**.

**Table 2:** Summary of parameters and formulae using data extracted from JIP-test.

Parameters	Explanation
*PI*_ABS=_RC/ABS [φP_o_/(1-φP_o_)] [ψ_o_/(1- ψ_o_)]	Performance index on absorption basis
S_m_ = (Area)/(*F*_m_ - *F*_o_)	Normalized total complementary area
ABS/RC=M_o_(1/V_J_) (1/φP_o_)	Average absorbed photon flux per PSII reaction center
ET_o_/RC =M_o_(1/V_J_) (1/V_J_)	Electron transport flux per RC (at t=*F*_o_)
DI_o_/RC =ABS/RC- TR_o_/RC	Dissipated energy flux per RC (at t=*F*_o_)
φP_o_=TR_o_/ABS= [1-(*F*_o_/*F*_m_)]	Maximum quantum yield of primary photochemistry
φD_o_=1-φP_o_=(*F*_o_/*F*_m_)	Quantum yield at t=*F*_o_ for energy dissipation
φE_o_=ET_o_/ABS= [1-(*F*_o_/*F*_m_)] ψ_o_	Quantum yield for electron transport (at t=*F*_o_)
Ψ_o_=ET_o_/TR_o_= (1-V_J_)	Probability that a trapped exciton moves an electron into the electron transport chain beyond Q_A_^-^ (at t=*F*_o_)
ABS/CS_m_≈ *F*_m_	Absorbed photon flux per cross section
TR_o_/CS_m_ =φp_o_ (ABS/CS_m_)	Maximum trapped exciton flux per cross section
ET_o_/CS_m_ =φE_o_ (ABS/CS_m_)	Electron transport flux from Q_A_ to Q_B_ per cross section
DI_o_/CS_m_ =(ABS/CS_m_) - (TR_o_/CS_m_)	Dissipated energy flux per RC (at t=*F*_m_)
RC/CS_m_ = φP_o_ (V_J_/M_o_) (ABS/CS_m_)	Probability that PSII Chl functions as an active center
δR_o_= RE_o_/ET_o_= (1−V_I_)/(1−V_J_)	Efficiency of electron movement from the reduced intersystem electron acceptors to the PSI end acceptors
φR_o_=RE/ABS =TR_o_/ABS (1 - V_I_)	Quantum yield for reduction of end electron acceptors at the PSI acceptor side

### 2.6 Calvin cycle key enzymes

Rubisco activity was measured spectrophotometrically following the method of Lilley and Walker (1974), with some modifications. The total activity was assayed after the crude extract had been activated in a 0.1 ml activation mixture containing 33 mM Tris-HCl (pH 7.5), 0.67 mM EDTA, 33 mM MgCl_2_ and 10 mM NaHCO_3_ for 15 min. Initial Rubisco activity measurements were carried out in 0.1 ml of reaction medium containing 5 mM HEPES-NaOH (pH 8.0), 1 mM NaHCO_3_, 2 mM MgCl_2_, 0.25 mM dithiothreitol (DTT), 0.1 mM EDTA, 1 U of glyceraldehyde 3-phosphate dehydrogenase, 0.5 mM ATP, 0.015 mM NADH_2_, 0.5 mM phosphocreatine, 0.06 mM ribulose-1,5-bisphosphate (RuBP), and 10 μl of extract. The change in absorbance at 340 nm was monitored for 90 s (YuPing et al., 2012). Activities of RCA, PGK, GAPDH, FBPase, and TK were measured using specific biochemical spectrophotometric kits (Yuchun Bio, China). SBPase activity was determined following the method of Harrison et al. (1998) method. FBA activity was measured using biochemical kits (COMIN Bio, China).

### 2.7 Quantitative real-time PCR

The total RNA was extracted from tomato leaves using the Trizol method. Primers used in this study were designed with Primer 6.0 based on the NCBI database (**Supplementary Table 2**). The tomato *actin* gene served as an internal control (Li et al., 2022a; Zhang et al., 2023). All primers were synthesized by Biotech Bioengineering Co. (Shanghai, China).

High-purity and integrity total RNA was reverse transcribed into cDNA using the Hyper ScriptTM III RT SuperMix for qPCR with gDNA Remover (NovaBio, China) following the manufacturer’s instructions. qRT-PCR amplification was conducted using the 2 x S6 Universal SYBR qPCR Mix (NovaBio, China). Real-time PCR was performed using a CFX96™ Real-Time PCR System (BIO-RAD, America), following the methods in the ChamQ Blue Universal SYBR qPCR Master Mix (Vazyme Biotech Co., Ltd, Nanjing, China). Three parallel replicates were prepared for each sample stored at -80°C, and biological replicates were performed for each gene. The 2^−ΔΔCt^ method Livak and Schmittgen (2001) was used to calculate the relative gene expression.

### 2.8 Statistical analysis

Data were analyzed using IBM SPSS 25 statistical software with one-way ANOVA. Differences’ significance was assessed using the Duncan test (P<0.05). Graphs were created using OriginPro 2023. Values in tables and graphs represent the mean ± standard deviation (SD). Three parallel replicates were prepared for each sample.

## 3 Results

### 3.1 Endogenous NO accumulation in tomato leaves under different treatments

**Figure 1:**
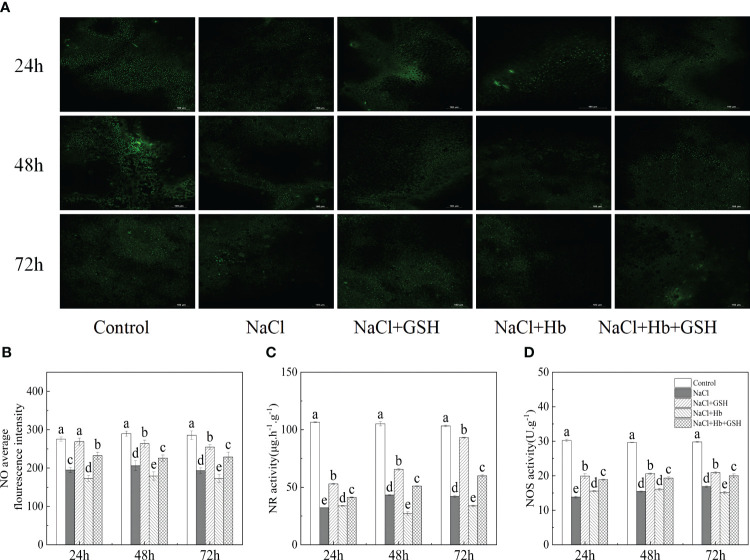
Effects of GSH (γ-glutamyl-cysteinyl-glycine), Hb (Hemoglobin, NO scavenger), Hb+GSH on NO fluorescence imaging **(A)**, average fluorescence intensity **(B)**, nitrate reductase (NR) activity **(C)**, and nitric oxide synthase (NOS) activity **(D)** in leaves of salt-stressed tomato seedlings. Notes: The green spots in figure A are the NO signal, and the short white line in the lower right corner is the 100-μm scale. Control, no added NaCl and sprayed with distilled water; NaCl, addition of 100 mM NaCl and sprayed distilled water; NaCl+GSH, added 100 mM NaCl, and sprayed 5 mM GSH; NaCl+Hb, with added 5 mM GSH. The results are presented as the mean ± SD (standard deviation) (n=3).Different lowercase letters represent significant differences, and the same lowercase letters represent no significant differences (p < 0.05, Duncan's range test).

As shown in **Figure 1**, in comparison with Control, the average NO fluorescence intensity, NR and NOS activity during the three measurement periods of NaCl treatment were significantly reduced by 41.3–42.7%, 59.1–69.9%, and 43.5–54.3%, respectively. Conversely, treatment with NaCl+GSH led to a significant increase in NO content, NR and NOS activity by 15.62–39.42%, 51.8–121.4% and 7.6–44.1%, respectively, compared to NaCl treatment. Treatment with NaCl+Hb resulted in a significant decrease in NO content and NR activity at 48 and 72 hours, as well as NOS activity at 72 hours. On the other hand, NaCl+Hb+GSH treatment significantly enhanced NO content, NR and NOS activity throughout the treatment period compared to NaCl+Hb treatment, with increases of 38.3–49.4%, 21.8–87.3% and 21.2–44.1%, respectively.

### 3.2 Photosynthetic gas exchange in tomato leaves under different treatments

**Figure 2:**
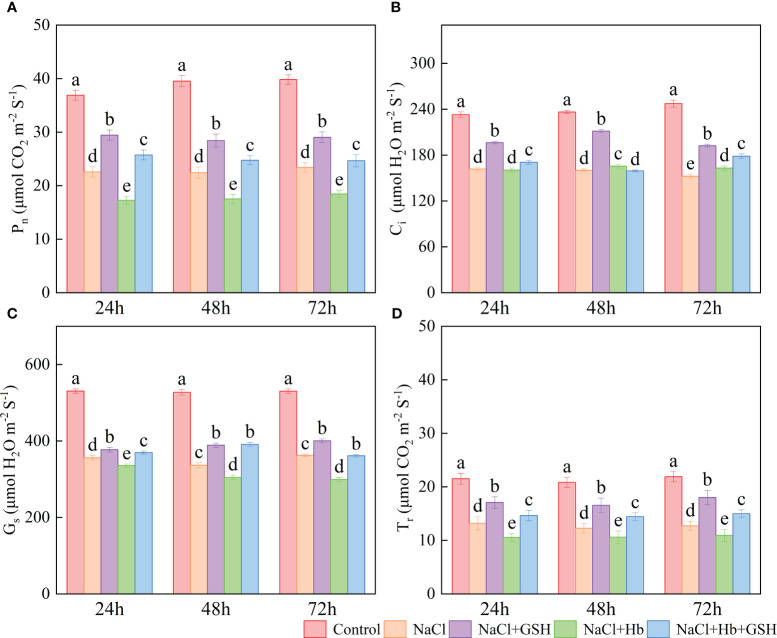
Effects of GSH, Hb, and Hb+GSH on the net photosynthetic rate (P_n_, **A**), intercellular CO_2_ concentration (C_i_, **B**), transpiration rate (T_r_, **C**), and stomatal conductance (G_s_, **D**) in leaves of salt-stressed tomato seedlings. Notes: Control, no added NaCl and sprayed with distilled water; NaCl, addition of 100 mM NaCl and sprayed distilled water; NaCl+GSH, added 100 mM NaCl, and sprayed 5 mM GSH; NaCl+Hb, with added 5 mM GSH. The results are presented as the mean ± SD (standard deviation) (n=3). Different lowercase letters represent significant differences, and the same lowercase letters represent no significant differences ( p < 0.05, Duncan's range test).

The application of NaCl significantly reduced the net photosynthetic rate (P_n_), stomatal conductance (G_s_), intercellular CO_2_ concentration (C_i_) and transpiration rate (T_r_) across all three treatment periods in comparison to Control (**Figure 2**). Conversely, the NaCl+GSH treatment led to a substantial increase in P_n_, C_i_, G_s_ and T_r_ values throughout the treatment period, showing enhancements 24.3–30.5%, 21.4–32%, 6.0–15.5% and 29.4–41.9%, respectively, when compared to the NaCl treatment. On the other hand, the NaCl+Hb treatment resulted in a notable reduction in P_n_, G_s_, and T_r_ throughout the treatment duration, while C_i_ values experienced at 48 and 72h. In comparison to the NaCl+GSH treatment, P_n_, G_s_ and T_r_ values over the treatment period, along with C_i_ at 24 and 72 h, exhibited significant decreases under NaCl+Hb+GSH treatment.

### 3.3 Impact of various treatments on the kinetic properties of transient fluorescence induction (JIP-test)

#### 3.3.1 OJIP curves

**Figure 3:**
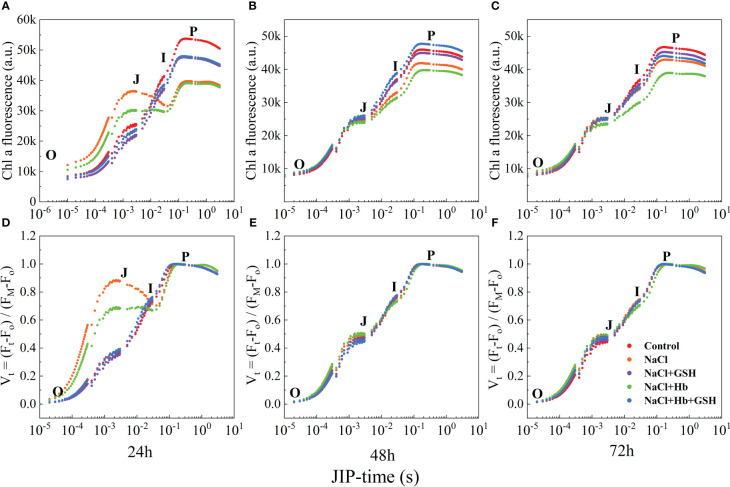
The fast chlorophyll a fluorescence induction (OJIP) curves **(A–C)** and normalized transients between O- and P-step expressed as V_t_ = [(F_t_ – F_o_)/(F_M_ – F_o_] **(D–F)** in leaves of salt-stressed tomato seedlings as affected by GSH, Hb, and Hb+GSH.

Under NaCl stress, the OJIP curves displayed deformations compared to Control, indicating a reduced amplitude of the I-P phase (**Figure 3A-C**). The entire curve flattened, and the amplitudes of the I and P phases, as well as the I-P phase of the OJIP curves, gradually decreased with prolonged treatment time. In contrast to the NaCl treatment, the shape of the OJIP curves underwent significant alterations during NaCl+Hb treatment, leading to a notable decrease in the amplitude of the I-P phase. However, the amplitudes of the I, P and I-P phases were significantly heightened throughout the three periods of NaCl+GSH treatment. However, the amplitudes of the I, P and I-P phases were significantly enhanced during the three periods of NaCl + GSH treatment. I-P phase amplitude significantly decreased, while the amplitudes of the I and P phases as well as the I-P phase were significantly higher in the three periods under NaCl+GSH treatment. Furthermore, the amount of phase I and P in OJIP curves treated with NaCl+Hb+GSH was lower than in those treated with NaCl+GSH during the three measurement periods. Normalization of the OJIP curves (**Figure 3D-F**) demonstrated a significantly higher J phase under NaCl stress compared with the control. This suggested an inhibition of Q_A_ to Q_B_ electron transfer on the PSII receptor side and a large accumulation of Q_A_^-^. Exogenous GSH application reduced the J phase under NaCl stress to varying degrees. The Hb application further increased the J phase under NaCl stress, weakening the effect of GSH. These findings indicate that GSH mitigated the partial damage to the PSII reaction center and decreased the ability of the PSII donor side to transfer electrons downstream under salt stress conditions, independently of NO.

**Figure 4:**
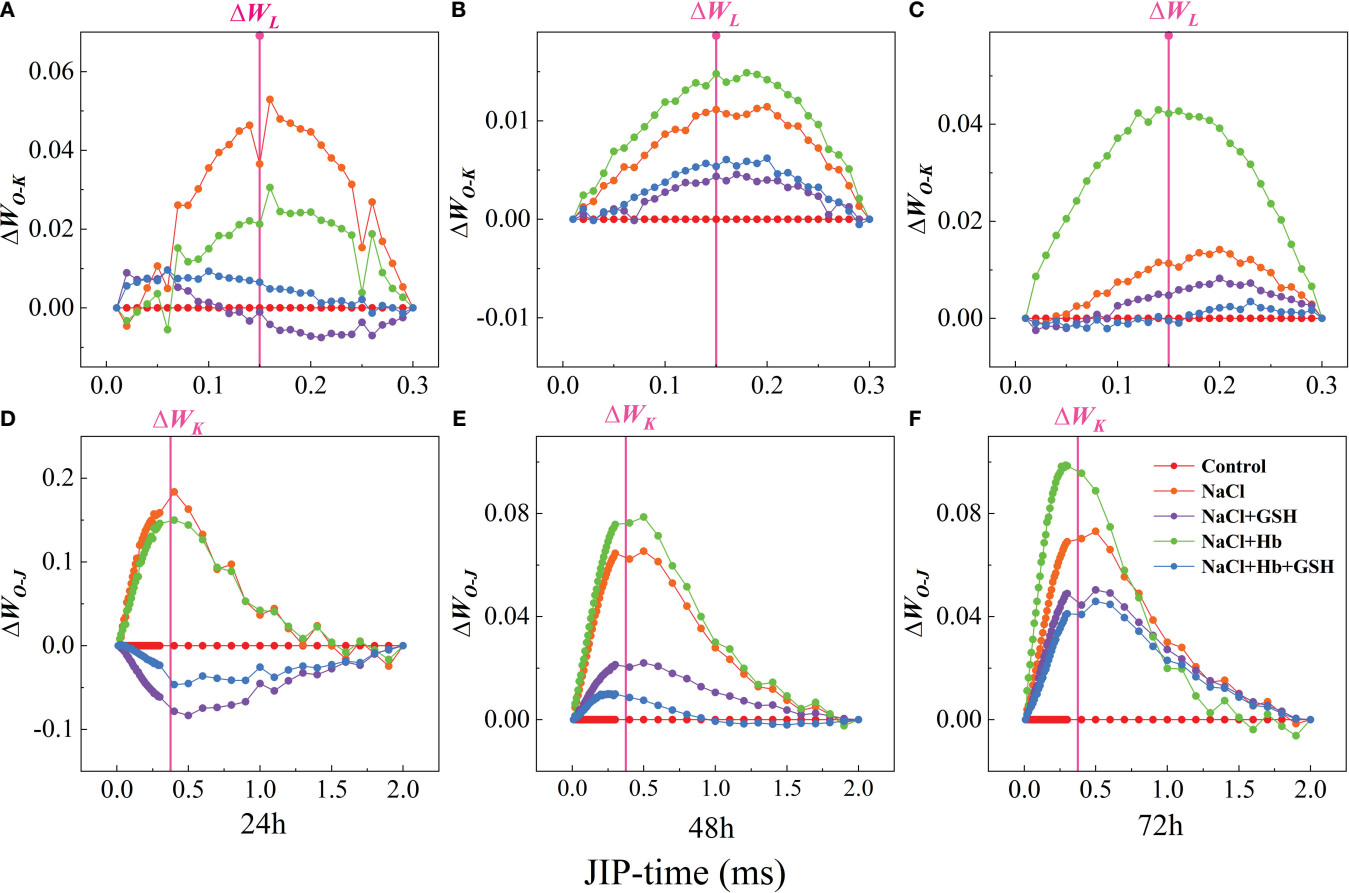
Difference kinetics ΔW_O-K_ = W_O-K(stress)_ - W_O-K(control)_ **(A–C)** in a linear time scale from 0 to 300 μs and ΔW_O-J_ = W_O-J(stress)_ - W_O-J(control)_ **(D–F)** in a linear time scale from 0 to 2 ms in leaves of salt-stressed tomato seedlings as affected by GSH, Hb, and Hb+GSH. ΔW_O-K_ = W_O-K(stress)_ - W_O-K(control)_ **(A–C)**, ΔW_O-J_ = W_O-J(stress)_ - W_O-J(control)_ **(D–F)**.

The presence of L and K-bands at 0.15 and 0.3 ms and the rise in ΔW*_L_* and ΔW*_K_* values were recognized as specific markers of vesicle-like dissociation and damage to the PS II donor-side complex (OEC), respectively. ΔW*_L_* at 24 and 72 h and ΔW*_K_* at 48 and 72 h were significantly higher under NaCl treatment compared to the control, while ΔW*_L_* and ΔW*_K_* were significantly lower under NaCl+GSH treatment compared with NaCl treatment to varying degrees (**Figure 4**). These results suggested that GSH application attenuates salt stress-induced vesicle dissociation and OEC damage. In addition, the Hb application further enhanced ΔW*_L_* at 48 and 72 h of salt stress treatment and ΔW*_K_* at the three measurement periods. The Hb application also enhanced ΔW*_K_* under NaCl+GSH treatment.

#### 3.3.2 JIP-test parameters

**Figure 5:**
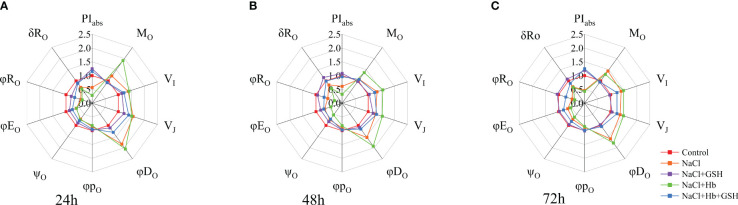
Spider plots were used to assess GSH, Hb, and Hb+GSH treatment influence on JIP-test parameters derived from chlorophyll a fluorescence OJIP transient curves **(A–C)** in salt-stressed tomato seedling leaves.

NaCl treatment had a significant impact on tomato plants compared to Control (refer to **Figure 5A-C**). There was a notable increase in the relative variable fluorescence (V_I_) at point I, the relative variable fluorescence (V_J_) at point J, the maximum rate at which Q_A_ was fully reduced (M_o_), the quantum ratio used for heat dissipation (φD_o_) in tomato seedling leaves. Furthermore, this increase was observed in the ratio of exciton-driven electron transfer by excitons captured by the PSII active reaction center (Ψ_o_), the quantum efficiency of the electron transfer from Q_A_^-^ to the electron transfer chain (φE_o_), the photosynthetic performance index (PI_abs_), the efficiency of electron transfer from Q_B_ to the PSI receptor side (δR_o_), the quantum yield of the terminal electron acceptor on the reduced PSI receptor side (φR_o_). These findings indicate that NaCl stress resulted in a decrease in the electron transfer capacity on the PSII receptor side and in the activation of the active PSII reaction centers. In contrast, NaCl+Hb treatment further significantly increased M_o_ and φD_o_, while significantly decreasing Ψ_o_, φE_o_, PI_abs_ and φR_o_. GSH application effectively alleviated M_o_ and φD_o_ increases under NaCl treatment to varying degrees and restored Ψ_o_, φE_o_, PI_abs_, δR_o_ and φR_o_ decreases to the control group levels (72 h) with extended treatment time. However, the Hb application weakened the effect of GSH on the above parameters under NaCl stress.

**Figure 6:**
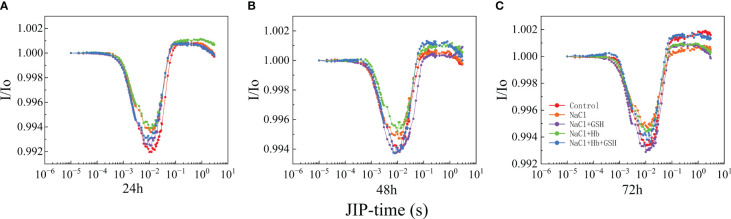
Curves of light-induced modulated 820 nm reflection kinetics (I/I_o_) **(A–C)** in leaves of salt-stressed tomato seedlings as affected by GSH, Hb, and Hb+GSH.

The 820 nm fluorescence reflectance kinetic curve (I/I_o_) is used to determine PSI complex activity due to adversity stress (**Figure 6A-C**), which demonstrated that NaCl stress could severely deform the leaf I/I_o_ curve compared to the control. This was expressed by the elevation of the lowest point of the descending phase and the decrease of the highest point of the ascending phase, suggesting that the PSI redox capacity was inhibited by salt stress. In contrast, the GSH application restored I/I_o_ curve deformation under NaCl treatment to varying degrees. The Hb application further heightened I/I_o_ curve deformation under NaCl stress and weakened the effect of exogenous GSH in alleviating the deformation.

#### 3.3.3 PSII reaction center activity and excited cross section phenomenological energy fluxes

**Figure 7:**
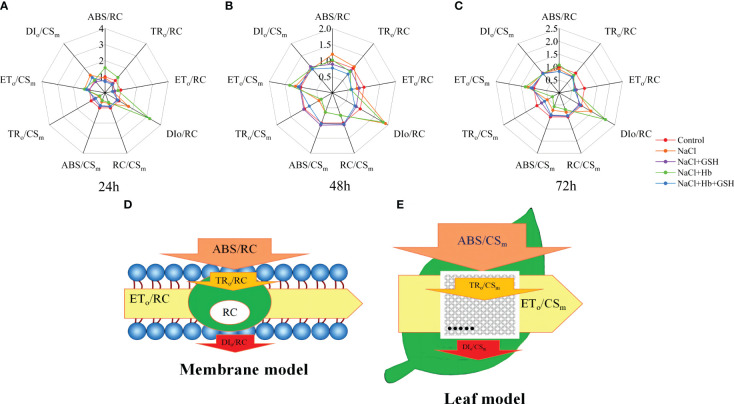
Spider plots of the energy distribution parameters per PSII reaction center (RC) and exciting cross-sectional area (CS_m_) **(A–C)** and the energy pipeline model of specific fluxes per RC **(D)** and phenomenological fluxes per excited CS_m_ **(E)** in salt-stressed tomato seedlings as affected by GSH, Hb, and Hb+GSH.

In tomato seedling leaves, the unit activity of the PSII reaction center (RC) showed a significant increase in the energy absorbed and thermally dissipated by RC (ABS/RC and DI_o_/RC), along with a notable decrease in the energy transferred by RC (ET_o_/RC) under NaCl treatment compared to the Control conditions (**Figure 7A-C**). Notably, NaCl+GSH treatment led to a significant increase in ET_o_/RC and a decrease in ABS/RC, DI_o_/RC and TR_o_/RC. Conversely, NaCl+Hb treatment increased ABS/RC, DI_o_/RC and TRo/RC values. Additionally, the ABS/RC, DI_o_/RC and TR_o_/RC values were significantly reduced in the NaCl+Hb+GSH treatment compared to the NaCl+Hb treatment. For the unit leaf cross-sectional area (CS_m_), the number of RCs (RC/CS_m_) and the energy absorbed and captured by the CS_m_ (ABS/CS_m_ and TR_o_/CS_m_) were significantly lower in the CS_m_ under NaCl stress compared to the Control. Although NaCl+GSH treatment significantly increased ABS/CS_m_, TR_o_/CS_m_, ET_o_/CS_m_ and RC/CS_m_, both NaCl+Hb and NaCl+Hb+GSH treatments significantly decreased ABS/CS_m_, TRo/CS_m_, ET_o_/CS_m_ and RC/CS_m_ values to varying degrees compared to NaCl and NaCl+GSH treatments (**Figure 7A-C**). The energy flux model is depicted in **Figure 7D, E**.

### 3.4 The role of NO in GSH-induced CO_2_ assimilation under salt stress

**Figure 8:**
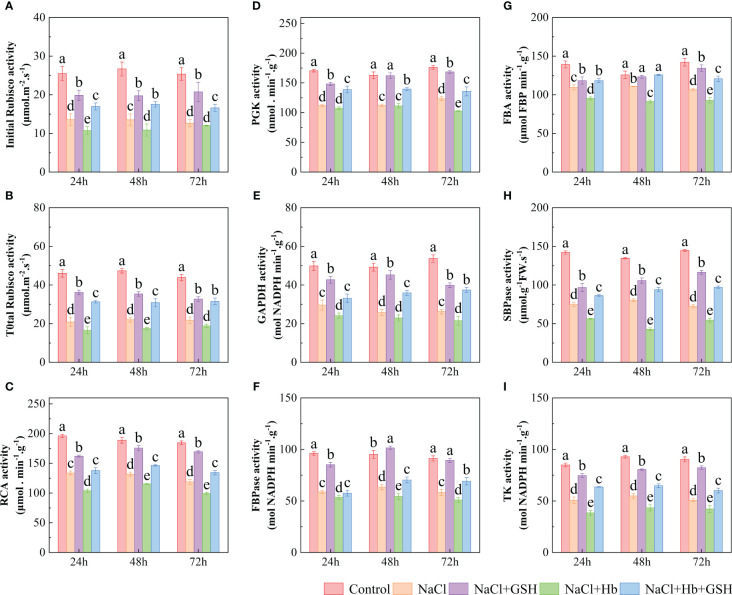
Effects of GSH, Hb, and Hb+GSH treatments on key enzymes of the Calvin cycle in leaves of salt-stressed tomato seedlings. Initial and total activity of Rubisco (**A, B**), RCA, PGK, GAPDH, FBPase, FBA, SBPase and TK **(C–I)**. Notes: Control, no added NaCl and sprayed with distilled water; NaCl, addition of 100 mM NaCl and sprayed distilled water; NaCl+GSH, added 100 mM NaCl, and sprayed 5 mM GSH; NaCl+Hb, with added 5 mM GSH. The results are presented as the mean ± SD (standard deviation) (n=3). Different lowercase letters represent significant differences, and the same lowercase letters represent no significant differences ( p < 0.05, Duncan's range test).

NaCl treatment significantly reduced Rubisco (both initial and total), RCA, GAPDH, PGK, FBA, FBPase, SBPase and TK activity by 46.7–50.1%, 50.3–54.6%, 11.9–24.9%, 40.6–51.1%, 30.1–35.7%, 29.9–34.5%, 33.3–38.8%, 40.5–49.9% and 40.2–43.8%, respectively (**Figure 8**). However, GSH application alleviated the inhibitory effect of NaCl stress, increasing above enzyme activity by 46.1–64.1%, 50.1–73.3%, 8.1–26.0%, 44.1–75.6%, 21.2–42.75%, 32.6–44.5%, 45.1–59.9% 29.1–60.7% and 46.5–62.1%, respectively. NaCl+Hb treatment significantly decreased TK activity, 24-hour RCA activity and 48-hour SBPase activity throughout the treatment period compared to NaCl stress. NaCl+Hb+GSH treatment significantly increased RCA, FBPase, FBA SBPase and TK activity, as well as PGK at 24 and 72 h and GADPH at 48 and 72 h compared to the NaCl+Hb treatment.

**Figure 9:**
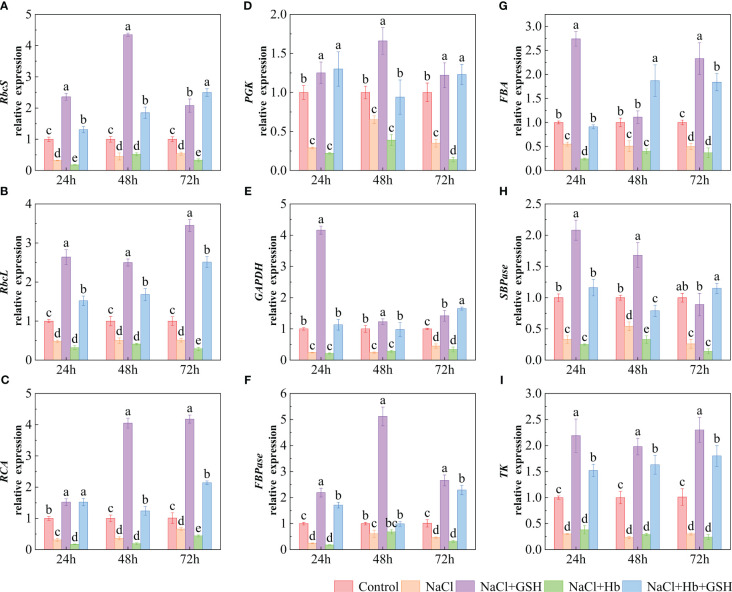
The relative expression of genes involved in the Calvin cycle in tomato seedling leaves under salt stress were analyzed for their response to GSH, Hb, and Hb+GSH. *RbcS*, *RbcL*, *RCA*, *PGK*, *GAPDH*, *FBPase*, *FBA*, *SBPase* and *TK* **(A-I)**. Transcript levels were quantified using quantitative PCR, which were then normalized to *actin* expression. Notes: Control, no added NaCl and sprayed with distilled water; NaCl, addition of 100 mM NaCl and sprayed distilled water; NaCl+GSH, added 100 mM NaCl, and sprayed 5 mM GSH; NaCl+Hb, with added 5 mM GSH. The results are presented as the mean ± SD (standard deviation) (n=3). Different lowercase letters represent significant differences, and the same lowercase letters represent no significant differences ( p < 0.05, Duncan's range test).

The transcript levels of key enzymes involved in CO_2_ carboxylation and reduction (*RbcS*, *RbcL*, *RCA*, *PGK* and *GADPH*) and enzymes related to RuBP regeneration (*SBPase*, *FBA*, *FBPase* and *TK*) were significantly downregulated under NaCl stress (**Figure 9**). However, NaCl+GSH treatment resulted in a significant upregulation of these genes. Conversely, NaCl+Hb treatment led to a significant downregulation of *FBA* at 24 h, *SBPase* at 48 h, *RbcL* and *PGK* at 72 h, *RbcS* and *RCA* at 24 and 72 h. No significant effects were detected on *GAPDH*, *FBPase*, *TK* transcript levels.

## 4 Discussion

Plant photosynthesis is essential for energy production and metabolism, and is highly susceptible to external environmental factors (Bahmani et al., 2019). P_n_ serves as a vital indicator of plant photosynthetic capacity (Simkin et al., 2019). During salt stress, the decrease in P_n_ can be attributed to both stomatal and non-stomatal limiting factors. In this investigation, the decline in P_n_ under salt stress coincided with reductions in C_i_, G_s_, T_r_ (**Figure 2**) and PI_abs_ (**Figure 5**). PI_abs_ is a comprehensive index that reflects photochemical efficiency. Previous studies have indicated that the reduction in PI_abs_ is more sensitive than F_v_/F_m_, accurately indicating the impairment of the plant’s photosynthetic machinery and the decrease in photochemical efficiency under adverse condition (Chen et al., 2021; Li et al., 2022b). The decrease in P_n_ due to NaCl stress was influenced by both stomatal-limiting and non-stomatal-limiting factors. These results were similar to the studies of *Hordeum jubatum* (Shi et al., 2021) and *Cucumis melo* (Sarabi et al., 2019). Furthermore, a correlation was noted between the reduction in P_n_ under salt stress and a decrease in NO signaling intensity, along with reduced NOS and NR activity. Conversely, the application of GSH under salt stress led to an improvement in P_n_, C_i_, G_s_ and PI_abs_, accompanied by an increase in NO levels as well as NR and NOS enzyme activity (**Figure 2**).

NO is widely distributed in various tissues and organs of plants, and exerts a positive regulatory role in plant growth and stress resilience. It is involved in the regulation of diverse physiological processes in plants (Fancy et al., 2017). Its cellular concentrations are decisive for its function, as a signaling molecule at lower concentrations, but triggers nitro-oxidative stress and cellular damage when produced at higher concentrations (Wani et al., 2021; Nishat Timilsina et al., 2022; Parveen et al., 2023). Salt stress-induced fluctuations in NO levels have been documented in different plant species, potentially influenced by salt type, stress intensity, duration and plant species. The results showed that external GSH improved P_n_ in salt-stressed tomato leaves by promoting the production of endogenous NO (**Figures 1**, **2**). To explore if external GSH influenced the photosynthetic capacity of tomato seedlings under salt stress through modulating endogenous NO levels, we treated tomato seedlings under salt stress with exogenous Hb, a NO scavenger. Numerous studies have previously demonstrated the ability of external Hb to efficiently decrease endogenous NO levels in plants (Shao et al., 2010; Lee and Hwang, 2015; Bahmani et al., 2019; Hu et al., 2023). In this investigation, the application of Hb not only resulted in a further reduction in P_n_ and endogenous NO levels under salt stress but also attenuated the beneficial impacts of GSH treatment, including the rise in P_n_ and endogenous NO levels. These findings strongly suggest that GSH mitigates the effects of NaCl stress by mediating endogenous NO levels, and is involved in regulating the decline of P_n_ in tomato plants. Hb application significantly reduced NR activity at 48 and 72 h and NOS activity at 72 h under salt stress to varying degrees, as well as NR and NOS activity under NaCl+GSH treatment. These results, similar to (Shao et al., 2010), suggested that Hb application also reduced endogenous NO levels via a down-regulation of the NO synthesis pathway.

Salt stress can significantly affect the photosynthetic apparatus, specifically the PSII located in the thylakoid lamellae, PSII is highly vulnerable to salt stress-induced photoinhibition, which can damage the overall photosynthetic efficiency (Zushi and Matsuzoe, 2017). The OJIP curve provides insights into PSII primary photochemical reactions, revealing the impact of environmental conditions on the photosynthetic apparatus, including the PSII donor and acceptor sides. The J-phase represented Q_A_^-^ rapid accumulation and increased J-phase fluorescence demonstrated a blockage in electron transfer from Q_A_ to Q_B_ on the PSII acceptor side. OJIP curve analysis provided insights into PSII functional status and environmental influence. The appearance of the K-band (ΔW*_K_* >0) before the OJIP curve rising to the J-phase reflected OEC damage on the PSII donor side. The appearance of the L-band with ΔW*_L_* > 0. On the other hand, indicated the dissociation of the basal vesicle-like bodies, resulting in an increased dissociation between the PSII complexes. V_J_ and V_I_ reflect the number of reaction centers in the J-phase and I-phase closure, respectively. An increase in V_J_ was a specific marker of blocked electron transfer from the PSII receptor side Q_A_ to the secondary quinone receptor Q_B_. However, an increase in V_I_ indicated a decrease in the ability of the PQ pool to accept electrons. In the current study, salt stress reduced the amplitude of the I-P phase and increased the ΔW*_K_*, ΔW*_L_*, and J phases (**Figures 3**, **4**), as well as elevated V_J_ and V_I_ values in tomato leaves (**Figure 5**). These changes indicated that salt stress damaged the PSII donor side OEC and increased dispersion between PSII complexes. This also led to the accumulation of Q_A_^-^ on the PSII acceptor side, resulting in the partial closure of PSII reaction centers. Furthermore, salt stress reduced Ψ_o_ and φE_o_ while promoting an increase in M_o_ (**Figure 5**). These results demonstrated a reduction in the openness of active PSII reaction centers and PQ pool capacity, as well as a decline in electron transfer ability from Q_A_ on the PSII acceptor side. GSH treatment however protected the PSII donor side OEC and reaction center structure under salt stress and also enhanced electron transfer and photochemical efficiency on the PSII acceptor side (**Figures 3**, **5**).

Conversely, the application of GSH shielded both the OEC and the reaction center structure on the donor side of PSII from salt stress, enhancing electron transfer and the photochemical efficiency of the PSII acceptor side (**Figures 3**, **5**). Furthermore, the use of Hb diminished the favorable outcomes of external GSH on electron transfer at the PSII donor side, the photosynthetic apparatus, and PSII acceptor measurements in NaCl-stressed tomato seedlings. Collectively, these findings indicate that NO is involved in mediating the advantageous effects of GSH on the structure and functionality of the PSII photosynthetic apparatus.

Under normal conditions, the PSII reaction center efficiently converts captured light energy into excitation energy. This energy is primarily utilized for carbon assimilation, while any surplus energy is dissipated as heat. However, under adversity stress, PSII reaction centers become temporarily inactive. Despite the continued absorption of light energy, PSII reaction centers fail to transfer it to the electron transport chain (Murchie and Niyogi, 2011). Additionally, the research illustrated a notable decline in RC/CS_m_, TR_o_/CS_m_ and ET_o_/CS_m_ in tomato leaves (**Figure 7**), indicating the degradation or deactivation of reaction centers and a reduction in light energy available for electron trapping and transfer (Li et al., 2017). The decrease in ABS/CS_m_ and the rise in DI_o_/CS_m_ under salt stress (**Figure 7**) indicated the activation of defense mechanisms in salt-stressed tomato plants, aligning with the observations of Yuan et al. (2014) findings. On the one hand, mitigating the overaccumulation of light energy by reducing the light energy absorption of PSII antenna pigments, and on the other hand, diminishing the buildup of excess excitation energy through the enhancement of the heat dissipation pathway. Moreover, salt stress induced an elevation in ABS/RC, TR_o_/RC and DI_o_/RC, while a decrease in RC/CS_m_ prompted a compensatory reaction. The remaining active RCs exhibited enhanced efficiency in absorbing, converting, and dissipating light energy, leading to improved consumption of excess energy in the electron transport chain (Chen et al., 2021). In contrast, GSH treatment not only mitigated the decrease in ABS/CS_m_, TR_o_/CS_m_ and ET_o_/CS_m_ in tomato leaves under salt stress, but also sustained elevated levels of DI_o_/CS_m_, ABS/RC, TR_o_/RC and DI_o_/RC (**Figure 7**). This protective mechanism optimizes energy allocation in PSII, preventing over-reduction of the photosynthetic electron transport chain and safeguarding the integrity and functionality of both the electron transport chain and the PSII reaction center.

In addition to plant leaf PSII, PSI reaction centers are vulnerable to adversity stress. The I/I_o_ curves reflect PSI primary photochemical reactions (Yuan et al., 2014). The fast descend phase in the I/I_o_ curves represents the oxidation of P_700_ and PC, the nadir point is the turning point of the redox of PSI. When the electrons from PSII promote PSI reduction, the I/I_o_ curve turns to a slow-rising phase, which represents the re-reduction of P_700_ and PC (corresponding to the I-P phase of the OJIP curve) (Guo et al., 2020). Therefore, the I/I_o_ curve is simultaneously affected by PSI and PSII activity and can reflect the coordination between these photosystems. The decrease in PSI activity prevents the PSII from transferring electrons to PSI, exacerbating the degree of PSII injury, meanwhile, PSI stability can ensure the rapid repair of damaged PSII (Yuan et al., 2014). The results of this study indicated that exogenous GSH effectively alleviated the reduction of PSI redox capacity in tomato plants under salt stress (**Figure 6**). Combined with the significant φR_o_ increase under NaCl+GSH treatment compared with NaCl treatment, it was hypothesized that GSH may enhance PSI activity via an increased ability or amount of electron transfer from PQ to P_700_^+^, facilitating PSI receptor-side electron transfer and coordinating the connectivity between PSII and PSI. However, the positive GSH effect on PSI in salt-stressed tomato leaves may be weakened by exogenous Hb application (**Figures 5**, **6**). This suggested that PSI activity regulation in salt-stressed tomato leaves by GSH required NO participation.

The Calvin-Benson cycle (CBC) is the primary CO_2_ fixation pathway utilized by C3 plants (Jensen et al., 2017). The CBC consists of the following three stages: CO_2_ fixation, CO_2_ reduction, and RuBP regeneration. Under adversity stress, the maintenance of high photosynthetic carbon assimilation capacity is a prerequisite for high yields (Zhao et al., 2021). Rubisco is the rate-limiting enzyme in CBC which plays a vital role in catalyzing CO_2_ fixation. The activity and expression of Rubisco directly influence the efficiency of CO_2_ carboxylation and the flow of electrons in the photosynthetic electron transfer chain (Sudhani and Moreno, 2015). *RbcL*, *RbcS*, and *RCA* genes encode the large subunit, small subunit and ribulose-1,5-bisphosphate carboxylase, respectively, which work closely together to regulate the structure and function of Rubisco holoenzymes. Many studies have reported that abiotic stresses (including low light, low temperature, drought, and other stresses) can negatively affect Rubisco and RCA activity and gene expression, affecting the photosynthetic rate of plants (Bi et al., 2017; Wijewardene et al., 2020). Sudhani and Moreno (2015) identified that GSH can convert Rubisco to an active state by modulating the RCA disulfide bond structure. Some reports have demonstrated that a high GSH/GSSG ratio increases the Rubisco activity and consequently CO_2_ assimilation via enhanced thiol and disulfide exchanges (Jiang et al., 2012). The authors’ previous study demonstrated that exogenous GSH can improve the CO_2_ carboxylation efficiency of tomato seedlings by up-regulating Rubisco and RCA activity as well as *RbcL*, *RbcS* and *RCA* expression under longer periods of salt stress (5, 10 and 15 d) (Liu et al., 2014). The results of the current study demonstrated that exogenous GSH treatment effectively counteracted the inhibitory effects of short-term salt stress (24, 48 and 72 h) on Rubisco activity, RCA activity, and *RbcL*, *RbcS*, and *RCA* expression. However, the beneficial effects of GSH on CO_2_ carboxylation efficiency under salt stress were diminished with the addition of exogenous Hb. These results suggested that exogenous GSH improved CO_2_ carboxylation and photosynthetic electron transfer efficiency in salt-stressed tomato plants, thereby alleviating photoinhibition caused by NADPH accumulation at the PSI receptor site. Furthermore, NO involvement in the positive regulation of GSH on CO_2_ carboxylation efficiency under NaCl stress was investigated. PGK and GAPDH are the main rate-limiting enzymes in the CO_2_ reduction phase (Zhang et al., 2011). The activity and gene expression of both directly affected CBC transport efficiency. SBPase (Ding et al., 2016), FBPase (Yu et al., 2020), TK (Bi et al., 2019) and FBA are the key enzymes in the RuBP regeneration stage. The strength of FBPase activity directly affects carbohydrate accumulation and photosynthetic efficiency. SBPase is located at the branching point between the assimilation and regeneration phases of the Calvin cycle, controlling carbon influx and regeneration. FBA catalyzes the cleavage of FBP into DHAP and G3P. These reactions play a role in plant responses to abiotic adversity such as salt stress (Lu et al., 2012). A reduction in TK activity decreases the efficiency of converting carbon-assimilated metabolites, leading to a decline in the photosynthetic rate of plants. *CsTK* antisense plants inhibit the growth of the capacity to assimilate carbon of *Cucumis sativus* under cold stress (Bi et al., 2019). This study demonstrated that exogenous GSH treatment effectively alleviated the inhibitory effects of NaCl stress on key enzyme activity and gene expression in the Calvin cycle (PGK, GAPDH, SBPase, FBPase, FBA and TK) (**Figures 8**, **9**). Hb application attenuated GSH effects to varying degrees. These results suggested that GSH enhanced carbon assimilation efficiency, relieved feedback inhibition of photosynthetic products, and promoted CBC operation, thereby mitigating the decline in carbon assimilation capacity caused by salt stress. Furthermore, NO was shown to be involved in the regulation of GSH on CO_2_ reduction and RuBP regeneration stages under salt stress.

## 5 Conclusion

In summary, the supplementation of exogenous GSH alleviated photoinhibition and enhanced photosynthetic efficiency in tomato seedlings subjected to NaCl stress. This was achieved through optimizing energy utilization in PSII reaction centers, safeguarding the structure and functionality of photosynthetic reaction centers, enhancing photochemical reactions and energy utilization efficiency, facilitating the operation of the Calvin-Benson Cycle, and alleviating the inhibitory effects of photosynthetic products. Moreover, the application of GSH resulted in an elevation of endogenous NO levels through the induction of the NO synthesis pathway. The presence of Hb attenuated the advantageous impacts of GSH on endogenous NO levels, as well as on the structure and functionality of the photosynthetic apparatus, and the efficiency of carbon assimilation in tomato seedlings under salt stress. These findings validated the role of NO in mediating the beneficial impacts of GSH in ameliorating photosynthesis in tomato seedlings under salt stress.

## Data availability statement

The original contributions presented in the study are included in the article/**Supplementary Material**. Further inquiries can be directed to the corresponding authors.

## Author contributions

YC: Conceptualization, Data curation, Methodology, Writing – original draft, Writing – review & editing, Investigation, Visualization, Formal analysis. XC: Writing – original draft, Writing – review & editing, Formal analysis, Investigation. JX: Investigation, Writing – original draft, Writing – review & editing, Formal analysis. XL: Investigation, Writing – original draft, Writing – review & editing. SP: Conceptualization, Writing – original draft, Writing – review & editing. HL: Conceptualization, Funding acquisition, Methodology, Project administration, Resources, Supervision, Writing – original draft, Writing – review & editing.
