# Supplementary material for: Nitric oxide signal is required for glutathione-induced enhancement of photosynthesis in salt-stressed Solanum lycopersicum L
Source: Front Plant Sci. 2024 Jun 17;15:1413653. doi: 10.3389/fpls.2024.1413653 (PMC11215142; doi:10.3389/fpls.2024.1413653)
Supplement: Supplementary file 2 [file Table_2.docx]

Supplementary Material

Nitric Oxide Signal is Required for Glutathione-Induced Enhancement of Photosynthesis in Salt-Stressed S*olanum lycopersicum* L.

Yundan Cong^a,b^, Xianjun Chen^c^, Jiayi Xing^a,b^, Xuezhen Li^a,b^, Shengqun Pang^a,b*^, Huiying Liu^a,b*^

^a^ Department of Horticulture, Agricultural College, Shihezi University, Shihezi, 832003, Xinjiang, P.R. China

^b^ Key Laboratory of Special Fruits and Vegetables Cultivation Physiology and Germplasm Resources Utilization of Xinjiang Production and Contruction Crops, Shihezi, 832003, Xinjiang, P.R. China

^c^ School of Life and Health Science, Kaili University, Kaili, 556011, Guizhou, P.R.China

*** Correspondence:**Huiying Liu E-mail:

[hyliuok@aliyun.com](mailto:hyliuok@aliyun.com)

Shengqun Pang E-mail：

[pangshqok@shzu.edu.cn](mailto:pangshqok@shzu.edu.cn)

## Supplementary Tables

**Supplementary Table 1.** Primer names and sequences used in qRT-PCR.

| Primer Names | Sequences | TM | Product length |
| --- | --- | --- | --- |
| Actin-F | TGGTCGGAATGGGAAAG | 55.16 | 191 |
| Actin-R | CTCAGTCAGGAGAACAGGGT | 57.77 |  |
| RbcL-F | CTGTATGGACCGATGGACTTAC | 58.02 | 119 |
| RbcL-R | AAGGTCTAAAGGGTAAGCTACATAAG | 58.21 |  |
| RbcS-F | TGAGACTGAGCACGGATTTG | 57.92 | 142 |
| RbcS-R | TTTAGCCTCTTGAACCTCAGC | 57.94 |  |
| *RCA*-F | TTGGACGGATTCTACATCGC | 57.78 | 204 |
| *RCA*-R | CTCCCCAAACACCCAAAATAAG | 57.46 |  |
| *PGK*-F | ACTCTTGTTAGCCATTTCAGTTTGT | 59.41 | 93 |
| *PGK*-R | ACCCTAAGAAGAATTCCAGAACA | 57.16 |  |
| *GADPH*-F | ACTCTGGTATATGTGTTACTC | 51.84 | 83 |
| *GADPH*-R | AGGGAAGCAAGATTACTAAA | 51.41 |  |
| *FBA*-F | ACATTCCGGCTCTTTTCAAAC | 57.36 | 125 |
| *FBA*-R | AGACCAACCCATTACAAGATCC | 57.76 |  |
| *SBPase*-F | AGAAATACACCTTGAGATACACCG | 58.34 | 150 |
| *SBPase*-R | TCAAGAATCCTAACGGTGCC | 57.6 |  |
| *FBPase*-F | AATTTCCATCTCTTCCCCACC | 57.61 | 139 |
| *FBPase*-R | TCGGTTTCTTGATCTGTGCTG | 58.58 |  |
| TK-F | TTGGAGAAGATGGACCTA | 51.06 | 141 |
| TK-R | GTGTCTTATTCTTGAGGATTG | 51.98 |  |

Note: actin: Actin gene; RbcL: Rubisco large subunit gene; RbcS: Rubisco small subunit gene; RCA: Rubisco activase gene; PGK: 3-Phosphogiyceric acid kinase gene; GADPH: glyceraldehyde-phosphate dehydrogenase; FBA: fructose 1,6-bisphosphate aldolase; SBPase: Sedoheptulose-1,7-bisphosphate gene; FBPase: Furetose-1,6-bisphosPhate phosphatase gene; TK: Transketolase gene.TM: melting temperature; Product length: the expected size of the amplicon.

**Supplementary Table 2.** Effects of GSH (γ-glutamyl-cysteinyl-glycine), Hb (Hemoglobin, NO scavenger), Hb+GSH on plant growth parameters of tomato seedlings grown under salt stress.

| Treatment | Time  (hour) | Shoot fresh weight  (g) | Root fresh weight  (g) | Shoot dry weight  (g) | Root dry weight  (g) |
| --- | --- | --- | --- | --- | --- |
| Control | 24h | 3.17±0.02a | 2.5467±0.07a | 0.2633±0.02a | 0.13±0.01a |
| NaCl |  | 3.1533±0.07a | 2.6033±0.05a | 0.2667±0.03a | 0.1167±0.02a |
| NaCl+GSH |  | 3.0733±0.17a | 2.57±0.1a | 0.26±0.02a | 0.1333±0.02a |
| NaCl+Hb |  | 3.0233±0.12a | 2.56±0.08a | 0.2433±0.03a | 0.12±0.01a |
| NaCl+Hb+GSH |  | 3.0233±0.11a | 2.6333±0.13a | 0.26±0.03a | 0.1233±0.01a |
| Control | 48h | 5.12±0.03a | 3.53±0.06a | 0.19±0.02a | 0.14±0.02a |
| NaCl |  | 4.89±0.16a | 3.54±0.07a | 0.21±0.01a | 0.12±0.02a |
| NaCl+GSH |  | 5.02±0.11a | 3.59±0.06a | 0.22±0.01a | 0.12±0.03a |
| NaCl+Hb |  | 4.95±0.1a | 3.54±0.0.12a | 0.19±0.02a | 0.11±0.01a |
| NaCl+Hb+GSH |  | 5.04±0.1a | 3.52±0.07a | 0.20±0.02a | 0.13±0.01a |
| Control | 72h | 6.85±0.06a | 4.11±0.08a | 0.22±0.01a | 0.14±0.01a |
| NaCl |  | 6.69±0.08a | 3.97±0.04a | 0.22±0.01a | 0.14±0.02a |
| NaCl+GSH |  | 6.94±0.13a | 4.06±0.08a | 0.22±0.01a | 0.13±0.03a |
| NaCl+Hb |  | 6.93±0.06a | 4.05±0.08a | 0.21±0.02a | 0.13±0.02a |
| NaCl+Hb+GSH |  | 6.90±0.04a | 4.10±0.15a | 0.22±0.04a | 0.12±0.03a |

Note: Value is mean±SD (n=3), and different letters in the same column with different treatments at the same treatment time indicate significant differences at 0.05 level between treatments.
